# On the contrast response function of adapted neural populations

**DOI:** 10.1152/jn.00413.2023

**Published:** 2024-01-24

**Authors:** Elaine Tring, Mario Dipoppa, Dario L. Ringach

**Affiliations:** ^1^Department of Psychology, David Geffen School of Medicine, University of California, Los Angeles, California, United States; ^2^Department of Neurobiology, David Geffen School of Medicine, University of California, Los Angeles, California, United States

**Keywords:** contrast response, mouse, population coding, power law, primary visual cortex

## Abstract

The magnitude of neural responses in sensory cortex depends on the intensity of a stimulus and its probability of being observed within the environment. How these two variables combine to influence the overall response of cortical populations remains unknown. Here we show that, in primary visual cortex, the vector magnitude of the population response is described by a separable power law that factors the intensity of a stimulus and its probability. Moreover, the discriminability between two contrast levels in a cortical population is proportional to the logarithm of the contrast ratio.

**NEW & NOTEWORTHY** The magnitude of neural responses in sensory cortex depends on the intensity of a stimulus and its probability of being observed within the environment. The authors show that, in primary visual cortex, the vector magnitude of the population response is described by a separable power law that factors the intensity of a stimulus and its probability.

## Graphical abstract

**Figure:**
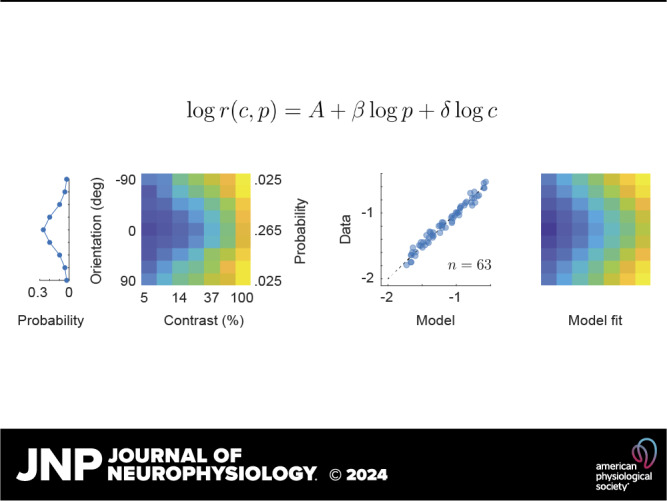

## INTRODUCTION

The responses of neurons in primary visual cortex to drifting sinusoidal gratings depend on their intensity, or luminance-contrast. The resulting contrast-response curves are often fit by the Naka-Rushton equation (1, 2), which when plotted as a function of the logarithm of the contrast, describes a symmetric, sigmoidal shape with a restricted linear range, while saturating at high and low contrast values (2–5). Here, motivated by theoretical models of contrast detection (6), we measure how the vector magnitude of the population response, defined as the Euclidean norm of the population vector ***r*** = (*r*_1_, *r*_2_, …, *r_n_*), which we denote by r=‖r‖, depends on stimulus contrast. As the shape of contrast response curves vary substantially from one neuron to the next (7), and some neurons in mouse V1 are suppressed by contrast (8, 9), it is not immediately clear how *r* should depend on stimulus contrast. Note the measurement of ***r*** is based on the responses of neurons in a population to the contrast of a fixed visual pattern. This pattern will be optimal for some cells in the population but not others. This approach differs from prior studies where contrast responses in individual neurons are measured using the optimal stimulus parameters for each neuron (2, 10–15).

In addition to stimulus contrast, the response of cortical neurons is strongly affected by the probability that a stimulus is encountered in any one visual environment through adaptation (5, 16–24). Given a finite set of stimuli, such as one comprised by sinusoidal gratings with different orientations, we define a specific “environment” as a probability distribution on the set. This probability distribution describes the likelihood any one stimuli will be observed within the environment. Different environments are defined by different distributions on the stimulus set. We have previously reported that the magnitude of the population response within an environment is linked to the probability that a stimulus is observed via a power law (25), *r* ∼ *p*^β^ (on the condition that *r* is approximately constant for a uniform distribution on the stimulus set). Here, *p* is the probability of the stimulus and the exponent β is negative, implying that the magnitude of the population response decreases as the probability of a stimulus increases.

Our goal in the present study is to investigate how the vector-magnitude of a population response depends jointly on stimulus contrast and probability. In other words, how does adaptation change the contrast response function of neural populations? To anticipate the results, we extend our previous findings by showing that response magnitude as a function of stimulus probability and contrast is described by a separable power law, *r* ∼ *p*^β^*c*^δ^. Here, *r* represents the magnitude of the response, *c* denotes stimulus contrast, *p* is the stimulus probability, and the exponents satisfy β < 0 and δ > 0. We discuss the implications of these results for the representation of contrast in adapted neural populations.

## MATERIALS AND METHODS

### Experimental Model and Subject Details

All experimental procedures were approved by University of California, Los Angeles (UCLA)’s Office of Animal Research Oversight (the Institutional Animal Care and Use Committee) and were in accord with guidelines set by the U.S. National Institutes of Health. A total of 9 mice, male (5) and female (4), aged P35–56, were used. These animals were obtained as a cross between TRE-GCaMP6s line G6s2 (Jackson Laboratory, https://www.jax.org/strain/024742) and CaMKII-tTA (https://www.jax.org/strain/007004). There were no obvious differences in the exponents of the power law between male and female datasets (Table 1, Wilcoxon test, *P* > 0.5 for both exponents, *n* = 14 experiments evenly split between male and female), thus we report our data together.

**Table 1.** Summary of the datasets

Animal	Type	Sex	ncells	Selected	gori25	gori50	gori75	beta_a	delta_a	r2_a	beta_r	delta_r	r2_r
adpt12_012_000	0	M	282	112	0.297	0.457	0.673	−0.358	0.574	0.966	−0.356	0.571	0.938
adpt14_001_000	0	M	487	133	0.241	0.390	0.579	−0.380	0.554	0.962	−0.376	0.561	0.906
adpt14_002_000	0	M	603	133	0.224	0.362	0.531	−0.301	0.497	0.925	−0.294	0.500	0.881
adpt15_001_000	0	F	724	155	0.231	0.361	0.515	−0.322	0.620	0.958	−0.317	0.616	0.927
adpt15_002_000	0	F	993	80	0.203	0.301	0.411	−0.252	0.522	0.946	−0.238	0.521	0.897
adpt15_020_000	0	F	356	95	0.234	0.382	0.558	−0.384	0.611	0.951	−0.372	0.602	0.902
adpt15_021_000	0	F	677	99	0.191	0.303	0.443	−0.204	0.437	0.941	−0.197	0.429	0.881
adpt16_010_000	0	F	86	23	0.210	0.364	0.513	−0.408	0.757	0.934	−0.377	0.772	0.836
adpt17_010_000	0	F	568	129	0.213	0.338	0.517	−0.345	0.817	0.978	−0.335	0.835	0.946
adpt17_050_000	0	F	821	257	0.260	0.398	0.570	−0.393	0.630	0.965	−0.375	0.620	0.928
adpt18_010_000	0	M	253	111	0.274	0.451	0.708	−0.431	0.640	0.957	−0.428	0.644	0.938
adpt18_030_000	0	M	548	156	0.246	0.392	0.559	−0.261	0.514	0.971	−0.257	0.512	0.940
adpt19_050_000	0	M	355	99	0.216	0.360	0.550	−0.345	0.697	0.973	−0.341	0.700	0.940
adpt19_060_000	0	M	482	106	0.194	0.310	0.483	−0.250	0.549	0.928	−0.235	0.547	0.862
adpt20_010_000	1	M	155	49	0.230	0.400	0.601	−0.421	0.707	0.962	−0.352	0.702	0.903
adpt20_020_000	1	M	230	67	0.217	0.364	0.578	−0.422	0.657	0.948	−0.232	0.657	0.899
adpt20_060_000	1	M	389	168	0.276	0.451	0.678	−0.336	0.661	0.966	−0.287	0.668	0.941
adpt21_010_000	1	F	519	213	0.345	0.491	0.612	−0.499	0.717	0.975	−0.404	0.685	0.938
adpt21_050_000	1	F	350	139	0.295	0.457	0.649	−0.391	0.651	0.940	−0.317	0.655	0.924

Each row corresponds to an independent experimental session. From left to right, the columns indicate: session name, type of experiment (0 = environments with the same von Mises distribution but different centers, 1 = natural orientation distributions), animal sex, total number of cells segmented, total number of cells that passed the data selection criterion, 25%, 50%, and 75% of one minus the circular variance of the responses, exponents estimated via the averaging procedure and the corresponding *R*^2^ value (Fig. 2*B*), the exponents estimated by modeling the ratios (*Eq. 1*) and the corresponding *R*^2^ values for this model (Fig. 2*F*).

### Surgery

Imaging was performed by visualizing activity through chronically implanted cranial windows over primary visual cortex. Carprofen was administered pre-operatively (5 mg/kg, 0.2 mL after 1:100 dilution). Mice were anesthetized with isoflurane (4–5% induction; 1.5–2% surgery). The core body temperature was maintained at 37.5°C. The eyes were coated with a thin layer of ophthalmic ointment during the surgery. Anesthetized mice were mounted in a stereotaxic apparatus using blunt ear bars placed in the external auditory meatus. A portion of the scalp overlying the two hemispheres of the cortex was subsequently removed to expose the skull. The skull was dried and covered by a thin layer of Vetbond. After the Vetbond dried (15 min), we affixed an aluminum bracket with dental acrylic. The margins were sealed with Vetbond and dental acrylic to prevent any infections. A craniotomy was performed over monocular V1 on the left hemisphere using a high-speed dental drill. Special care was taken to ensure that the dura was not damaged during the process. Once the skull was removed, a sterile 3 mm diameter cover glass was placed directly on the exposed dura and sealed to the surrounding skull with Vetbond. The remainder of the exposed skull and the margins of the cover glass were sealed with dental acrylic. Mice were allowed to recover on a heating pad and once awake, they were transferred back to their home cage. Carprofen was administered post-operatively for 72 h. We allowed mice to recover for at least 6 days before the first imaging session.

### Two-Photon Imaging

We conducted imaging sessions in awake animals starting 6–8 days after surgery. Mice were positioned on a running wheel and head-restrained under a resonant, two-photon microscope (Neurolabware, Los Angeles, CA) controlled by Scanbox acquisition software and electronics (Scanbox, Los Angeles, CA). The light source was a Coherent Chameleon Ultra II laser (Coherent Inc., Santa Clara, CA). The excitation wavelength was set to 920 nm. The objective was an x16 water immersion lens (Nikon, 0.8 NA, 3 mm working distance). The microscope frame rate was 15.6 Hz (512 lines with a resonant mirror at 8 kHz). The field of view was 730 µm × 445 µm. The objective was tilted to be approximately normal on the cortical surface. Images were processed using a standard pipeline consisting of image stabilization, cell segmentation, and signal extraction using Suite2p (https://suite2p.readthedocs.io/) (26). A custom deconvolution algorithm was used (27). We have previously compared the results of different deconvolution algorithms and found little variability in the results (25). A summary of the experiments, including statistical summaries, is presented in Table 1.

### Visual Stimulation

We used a Samsung CHG90 monitor positioned 30 cm in front of the animal for visual stimulation. The screen was calibrated using a Spectrascan PR-655 spectro-radiometer (Jadak, Syracuse, NY), generating gamma corrections for the red, green, and blue components via a GeForce RTX 2080 Ti graphics card. Visual stimuli were generated by a custom-written Processing 4 sketch using OpenGL shaders (see http://processing.org). At the beginning of each experiment, we obtained a coarse retinotopy map of primary visual cortex as described elsewhere (25). The center of the aggregate population receptive field was used to center the location of our stimuli in these experiments. Stimuli were presented within a circular window with a radius of 25°. The spatial frequency of the gratings was fixed at 0.04 cpd. The orientation domain was discretized in steps of 10°, from 0° to 180°. The spatial phases at each orientation were uniformly randomized from 0° to 360° in steps of 45°. Contrast was drawn uniformly from a set 5% × ζ*^q^* for *q* = 0, …, 6 and ζ = (100/5)^(1/6)^ ≈ 1.6475 A total of 5,400 trials (6 blocks of 5 min each at 3 stim per second) were collected for each environment. For a uniform environment, this results in an average of 300 trials per orientation. The appearance of a new stimulus on the screen was signaled by a TTL line sampled by the microscope. As a failsafe, we also signaled the onset of the stimulus by flickering a small square at the corner of the screen. The signal of a photodiode was sampled by the microscope as well. In some experiments (Fig 3*C*) the orientation distributions were drawn from natural samples we measured at UCLA as described in a prior study (25).

### Optimal Stimulus-Response Delay

For each environment, we calculated the magnitude of the population response *T* microscope frames after the onset of the stimulus, where *T* ranged from −2 to 15. The frame rate of the microscope was 15.53 frames/s. The time to peak of these curves is agreed for all environments. We therefore averaged the magnitudes across all the three environments and defined the optimal stimulus-response delay as the time (in microscope frames) between the onset of the stimulus and the peak response magnitude of the population (averaged across orientations and contrasts). When computing the mean population vector for a given stimulus, we also averaged across spatial phases, thus minimizing the effect of eye movements on our analyses.

### Statistics and Reproducibility

We conducted experiments by independently measuring the adaptation of neural populations in visual cortex in 19 different instances (see Table 1). Linear models were fitted to the data using Matlab’s fitlm function. The goodness of fit of linear models was evaluated using the *R*^2^ statistic [the coefficient of determination (28)]. Both coefficients were highly significant in all experiments (*p* values less than 10^−6^). As the study did not involve different groups undergoing different treatments, there was no need for randomization or blind assessment of outcomes. Data selection was used to select a subset of neurons with good orientation tuning, attaining a circular variance (29) of less than 0.5. Cohen’s *d* was computed using Matlab’s meanEffectSize function for different contrasts levels for a given environment and orientation. We then computed the average discriminability by pooling the data across all orientations and environments.

## RESULTS

**Figure 1.**
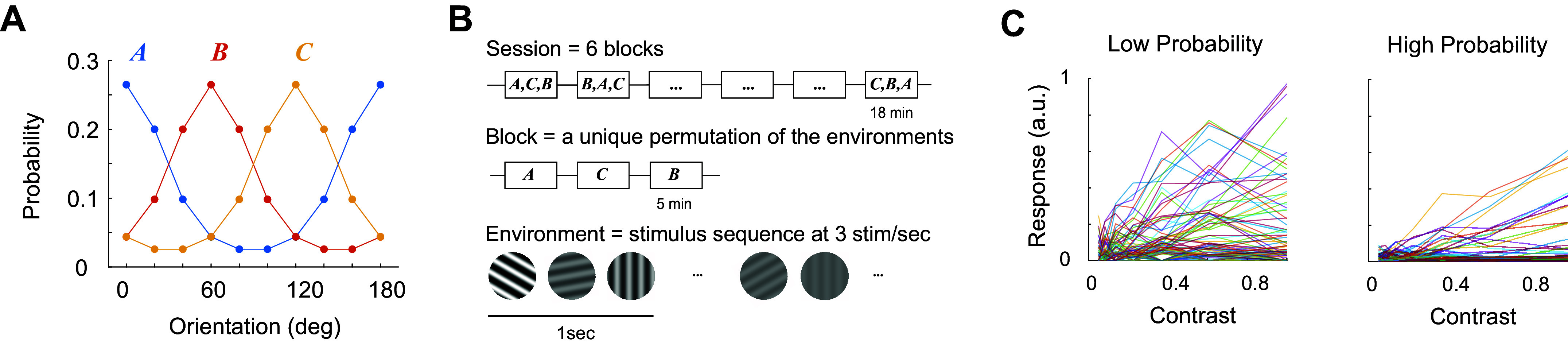
Experimental protocol. *A*: sessions included the presentation of three environments, *A*, *B*, and *C*. Different probability distributions describe the likelihood any one stimuli will be observed within each environment. In our initial set of experiments, three environments were defined by associating them with von Mises distribution in the orientation domain centered at 0°, 60°, and 120°, respectively. The contrast of any selected grating was drawn uniformly from a set of seven contrast levels equally spaced on a logarithmic scale from 5% to 100%. *B*: A session consisted of six blocks, each containing a unique permutation of all three environments. Each environment was presented for 5 min by independently drawing stimuli according to its probability distribution at a rate of 3 per second. A blank screen was presented for 1 min between environments. From one session to the next, the order of the permutations was randomized. Stimuli were presented through a 20 deg circular window centered on the aggregate receptive field of the population (see methods). *C*: examples of a contrast response functions in a population of neurons to a stimulus of a fixed orientation in an environment where it appeared with low probability (*left*) and when it appeared with high probability (*right*).

We recorded from excitatory neurons in layer 2/3 in mouse primary visual cortex (area V1) using in vivo, two-photon imaging (methods). We measured neural responses to the presentation of a sequence of rapidly flashed, sinusoidal gratings with orientations drawn from von Mises distributions representing three, different statistical environments, with peaks centered at 0°, 60°, and 120° (the concentration of the distribution, *κ* = 1.2, was constant across environments) (Fig. 1). For each orientation, the contrast was drawn uniformly from a set of seven values logarithmically spaced between 5% and 100%. The raw measurements represent the contrast response of individual neurons for stimuli with different orientations and in different environments. The shapes of the individual contrast response functions to a fixed stimulus are diverse. In an environment where the stimulus appeared with low probability some contrast responses saturate, others are nonmonotonic (peaking at intermediate contrasts), in several instances, responses appear almost linear, and many other cells in the population, for which the stimulus was nonoptimal, yielded weak responses (Fig. 1*C*, *left*). The response of the same population, when the same stimulus appeared with high probability in the environment, yielded responses that tended to be linear (Fig. 1*C*, *right*).

Instead of analyzing the changes in individual contrast response functions, which look rather complex, can the behavior of the population response be characterized in a simple manner? We analyzed the data by computing, for each environment, the average magnitude of the population response as a function of contrast and orientation. We then averaged the responses across environments after aligning them to the peak of the orientation distribution. This step relies on the distributions across environments being the same except for the location of their peaks (we later describe how to relax this condition). Such averaging minimizes variability due to tuning inhomogeneities in a population, where the norm of the population depends on stimulus orientation when the probability distribution is uniform (30). Finally, each orientation can be associated with its probability of appearance. The resulting data set can be interpreted as describing how the magnitude of the population response changes as a function of stimulus strength (its contrast) and stimulus probability, *r*(*c*, *p*).

**Figure 2.**
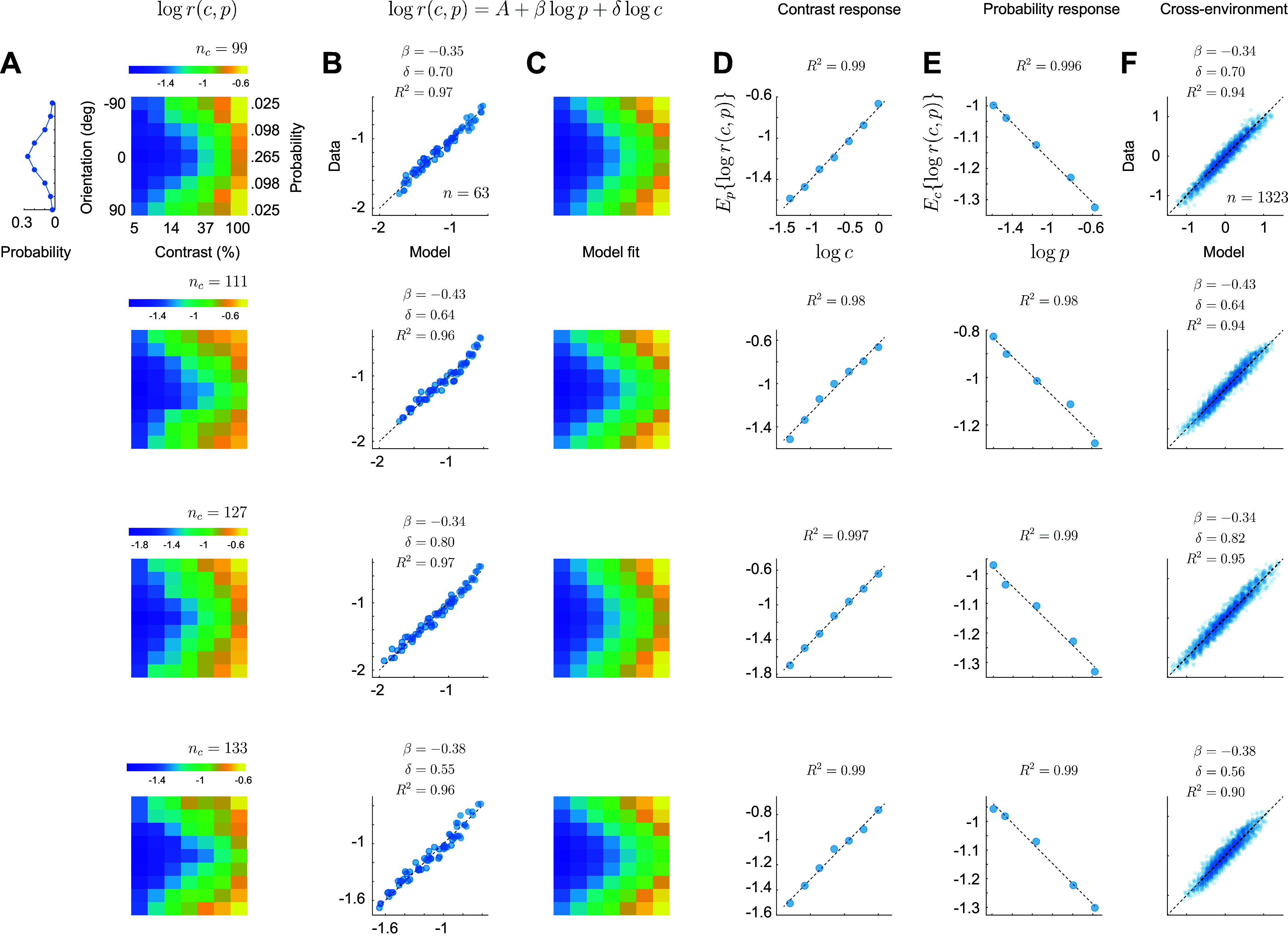
Cortical populations combine stimulus probability and intensity via a power law. Each row of panels describes the outcome of a different experimental session. Unless noted otherwise, the labels and ranges of the axes on the top row apply to all others. *A*: empirical dependence of response magnitude with stimulus probability and contrast. The pseudocolor image represents the vector-magnitude of the population in a logarithmic scale (scalebar). The number of cells in the population is shown by the value of *n_c_*. The contrast of stimuli varies along the *x*-axis, while the probability (or orientation) varies along the *y*-axis. The results of the three environments were averaged by aligning the peak of the probability distribution with 0°. The distribution of orientations (after alignment) is shown by the graph on the left. *B*: fits of the model *r* = *A P*^β^c^δ^ to the data. The *x*-axis represents the fits of the model and the *y*-axis the data. The exponents estimated appear at the inset. The value of *n* represents the number of data points. The goodness of fit, *R*^2^, is higher than 0.95 in all cases, indicating the quality of the model. *C*: the fits shown in *B* now represented as in the same format as the data in *A*. *D*: average contrast response of the population obtained by averaging the rows of the images in *A*. The data falls approximately on a line in log-log coordinates. *E*: average probability response of the population obtained by averaging the columns of the images in *A*. The data falls approximately on a line in log-log coordinates. *F*: modeling the ratio population responses across environments as the product between a power of the ratio of the stimulus probabilities and a power of the ratio of the stimulus contrasts. The data are well fit by a line in log-log coordinates. The estimated exponents and goodness of fit appear at the inset. In these and all subsequent analyses the logarithm is base 10.

Measurements of *r*(*c*, *p*) can be visualized as a 2-D pseudocolor image (Fig. 2*A*). Each row in the image describes how the magnitude of the response changes with contrast. Each column describes how the response changes with the probability of the stimulus (the peak of the orientation distribution is centered at 0° and the data averaged across environments). Note that in these environments different orientations can appear with the same probability (we test more complex environments below) (Fig. 2*A*, *top left* distribution). As expected, the magnitude of population response decreases with the increasing probability of a stimulus (adaptation), and the response increases with contrast. How can the surface *r*(*c*, *p*) be described in terms *c* and *p*? We discovered that a three-parameter model of the form *r* = *A p*^β^ *c*^δ^ fits the data well. To fit the model, we first take logarithms, resulting in a linear model, log *r* = log *A*+ β log *p* + δ log *c*, with high-quality fits (*R*^2^ = 0.95 ± 0.02, mean ± 1SD, Fig. 2, *B* and *C*). The model fits (Fig. 2*C*) are symmetric around the horizontal axis because the probability of the stimulus is symmetric as well. The fitted coefficient β is negative (−0.34 ± 0.07, mean ± 1SD, *n* = 14), meaning that the higher the probability of a stimulus, the lower the response (summary statistics in Supplemental Table S1). The coefficient δ is positive (+0.59 ± 0.10, mean ± 1SD, *n* = 14), meaning that the higher the contrast of a stimulus, the higher the response. As a result of the separability of the model, stimulus contrast and probability trade off against each other in a simple way – a twofold increase in the probability of a stimulus can be countered by increasing its contrast by a factor of 2^−(β/δ)^ ≈ 3.33 leaving the response magnitude invariant. One would be justified in saying that the log-probability of a stimulus and its log-contrast trade in the same “currency.” Note that the value of the estimated parameter A will depend on details of how the spike inference signal is normalized and not critical in demonstrating the power law behavior.

A more general method of analysis that avoids averaging the data across environments consists of modeling how the ratio of responses between environments changes as a function of the ratio of contrasts and the ratio of probabilities for each stimulus. Given an environment *X*, we denote by *r*(*c_X_*, *p_X_*(θ)) the average response to a grating with orientation θ, contrast *c_X_*, and probability *p_X_*(θ). Then, our analyses show that the ratios of response magnitudes between two environments, *X* and *Y*, can be described by extending our prior model (25):

(*1*) $$\frac{r\left(c_{X},p_{X}\left(\theta \right)\right)}{r\left(c_{Y},p_{Y}\left(\theta \right)\right)}={\left(\frac{c_{X}}{c_{Y}}\right)}^{\delta }{\left(\frac{p_{X}\left(\theta \right)}{p_{Y}\left(\theta \right)}\right)}^{\beta }$$

Taking logarithms results in a linear model we can fit to estimate the values for the exponents. The population responses averaged across all orientations show a linear dependence with contrast in log-log coordinates (Fig. 2*D*), illustrating the main effect of contrast. Interestingly, the population response does not saturate at low or high contrasts (within the range tested). Similarly, the population response average across all contrasts shows a linear dependence with the probability of a stimulus (Fig. 2*E*), showing the main effect of stimulus probability. Finally, the full model can be evaluated by plotting scatterplots between the measured ratios (taking pairwise combinations of the three environments and all stimulus orientations) and their linear fits in log-log coordinates (Fig. 2*F*).

**Figure 3.**
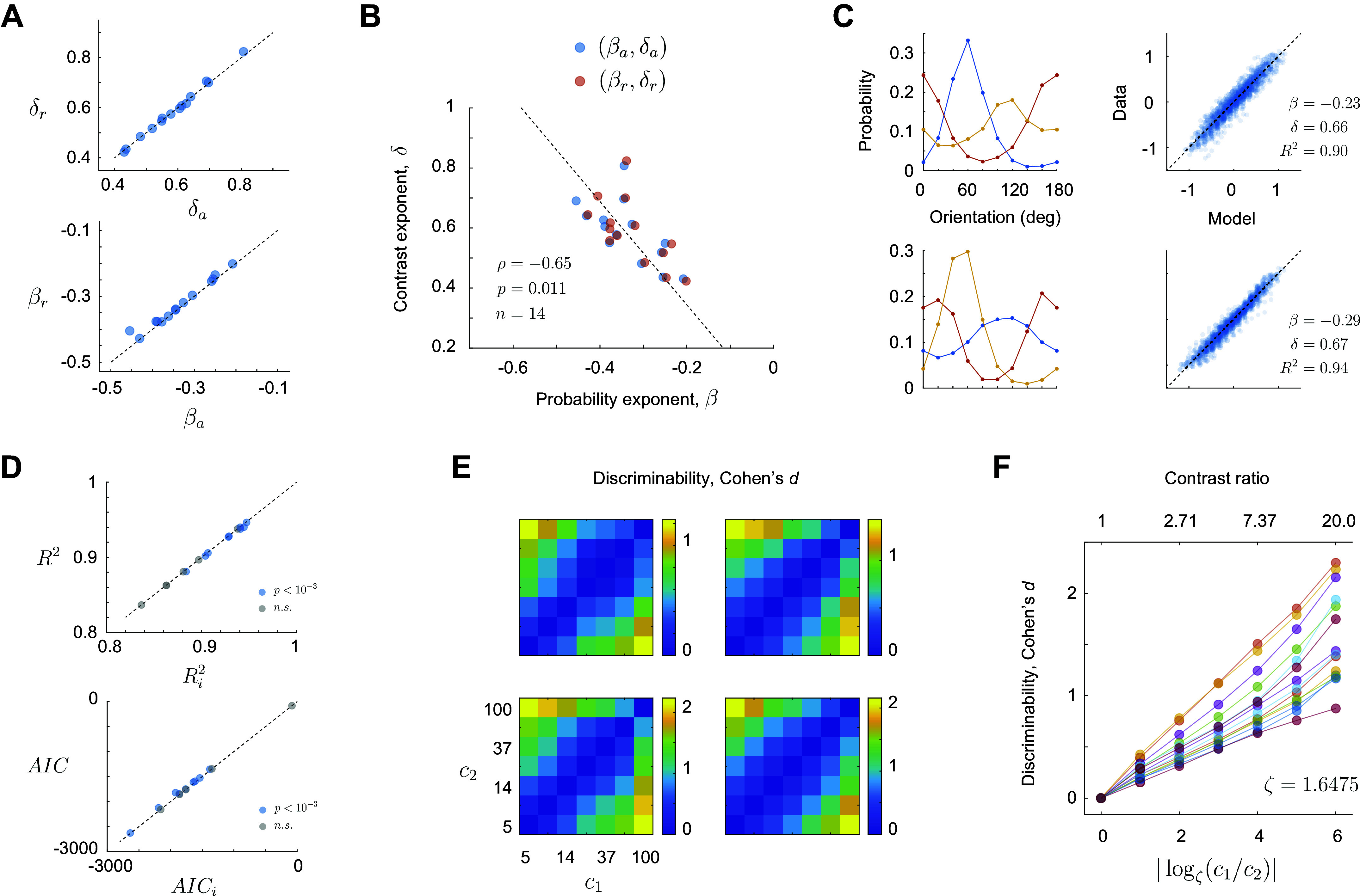
Additional properties of the power law. *A*: estimates of the exponents agree for the two methods employed: averaging across environments or modeling the ratio of responses across environments. The dashed line is the identity line. *B*: there is a significant, negative correlation between the exponents. Dashed line is the best linear fit. *C*: modeling the ratio of population magnitudes between environments via a power law holds when the stimulus distributions differ across environments. Each row of panels shows the result from different experiments. *D*: goodness of fit for models with and without an interaction term. *Top panel* shows the coefficient of determination for a linear model without interaction (*R*^2^) and with interaction ($R_{i}^{2}$). The blue data points indicate experiments where the fitted coefficient of the interaction term was statistically significant at the 0.001 level. Gray dots correspond to not significant cases. The *bottom panel* shows the Akaike’s Information Criterion for the linear model without interaction (AIC) and with an interaction term (AIC*_i_*). For the AIC more negative values represent better models. *E*: four examples showing the dependence of discriminability (Cohen’s *d*) between gratings of the same orientation at two contrasts levels (*c*_1_ and *c*_2_). *F*: average discriminability is approximately linear with the logarithm of the ratio between the contrasts to be discriminated. The set of contrasts used are spaced logarithmically from 5 to 100% in six steps. The ratio between adjacent contrasts is ζ = (100/5)^(1/6)^ ≈ 1.6475. Thus, we found convenient to express the logarithm represented along the *x*-axis in base *ζ.*

It is reassuring that the estimates of β and δ obtained by fitting the data averaged across environments (β*_a_* and δ*_a_*) or by fitting the ratios across environments (β*_r_* and δ*_r_*) are nearly identical (Fig. 3*A*). Moreover, the exponents β and δ are significantly anti-correlated and well fit by the relationship, δ ≈ −1.72β (ρ = −0.65, *P* = 0.011) (Fig. 3*B*). This correlation means that the more “linear” the population contrast response function is (that is, the closer the value of δ is to +1), the stronger the population adapts (its exponent β is closer to perfect adaptation, attained for β = −1). The relationship linking ratios across environments (*Eq. 1*), is rather general and applied in scenarios where the shape of the distribution differed from one environment to the next (Fig. 3*C*). These distributions were obtained from empirical distributions of orientation in natural image patches (25). To evaluate if the addition of an interaction term in *Eq. 1* would improve the quality of the fits we plotted the *R*^2^ values and Akaike’s Information Criterion (AIC) for models with and without the interaction term (Fig. 3*D*). The data fall largely on the main diagonal, indicating that the interaction term provided only minor improvements even when the interaction was statistically significant at the *P* < 0.001 level (Fig 3*D*, blue dots). The average *R*^2^ for the models without interaction was *R*^2^ = 0.9086 ± 0.0339 (mean ± 1SD) and adding the interaction term increased merely to $R_{i}^{2}=0.9099\pm 0.0343$ (mean ± 1SD). This difference was not significant (rank-sum, signed test, *P* = 0.30).

Finally, we examined how the discriminability between two gratings (of equal orientation) depends on their contrasts *c*_1_ and *c*_2_ (Fig. 3, *E* and *F*). We found that discriminability, computed as Cohen’s *d* is proportional to the logarithm of *c*_1_/*c*_2_ (it is convenient to take the base of the logarithm equal to the ratio between adjacent contrasts in our experiments, ζ = 1.6575) (see methods). Each of our experiments generated an approximate relationship *d* ∼ *k*|log_ζ_ *c*_1_*/c*_2_| (Fig. 3*F*), but the slope *k* varied between experiments (mean 0.25 ± 0.07, mean ± 1SD). This result is puzzling as it implies the population obeys a neurometric Weber’s law when, in humans, contrast discrimination thresholds violate it (31). Unfortunately, to our knowledge, there are no measurements of behavioral, suprathreshold contrast discrimination in the mouse to compare our data to at present.

## DISCUSSION

We have shown that a simple model, log *r* = log *A* + β log *p* + δ log *c*, accounts for the average magnitude of population responses as a function of stimulus contrast and probability (Fig. 2, *A*–*C*). The data showed no strong interaction between the two terms (Fig. 3*D*), allowing stimulus contrast and probability to trade off against each other. The model implies that, in any given state of adaptation, the population contrast response function is just a line in log-log coordinates, which does not saturate within the range of contrasts tested. Adaptation at the level of single cells can often be described as a shift in the semi-saturation constant. At the population level, the description is even simpler – changes in adaptation merely translate the contrast response function vertically in log-log coordinates (by the term β log *p*). A more general model (*Eq. 1*) extends prior findings (25) and accounts for the data without the need of averaging across environments (Fig. 2*F*). An important, open question is what kind of normative models may predict the power law behavior observed. Some recent theoretical proposals of how adaptation may affect the cortical representation based on notions from efficient coding (32–34) and mechanistic, models of V1 (35–39) that aim to capture how the V1 architecture shapes cortical responses, could readily be tested to see if they are consistent with our present findings.

The contrast response of V1 neurons has been studied extensively before, but a direct comparison to our findings is precluded due to differences in the overall experimental approach. Here, we define the contrast response of the population as the dependence of the population Euclidean norm, r=‖r‖, with contrast for a fixed stimulus pattern. The entries in the vector ***r*** represent the unnormalized responses of individual neurons. Instead, in most prior studies, the contrast response function was measured after optimizing stimulus parameters (such as spatial frequency, temporal frequency, and size) for each individual neuron (2, 10–15). In one instance where this was not the case, population responses were based on multi-unit activity and further averaged across different cortical sites according to their preference for orientation (40). In earlier work, the contrast responses across a group of neurons were normalized to their maximum or mean before being averaged to yield a population contrast response (37, 40). We note, the average contrast response of a population is not necessarily the same as the dependence of the population norm on contrast (as the *l*_2_ of the population vector is not the same as the *l*_1_ norm).

Altogether, our findings reveal that a simple relationship accounts for the interplay between stimulus strength and probability in driving the magnitude of population responses. To verify that non-linearities in calcium imaging (41) are not distorting our main findings, we are replicating these experiments using Neuropixels probes. One additional caveat is that the data and the model, as developed so far, are limited to situations where the distribution of contrasts in the environment is independent of stimulus orientation. Relevant scenarios exist where this is not the case. In cases of astigmatism, for example, the contrast of gratings at some orientations is reduced compared to others. Future work will examine how the cortex adapts under such conditions with health relevance. Finally, we note that we have yet to analyze how the direction of the population vectors change with contrast and stimulus probability. The fact that contrast response functions are heterogenous (Fig. 1*C*) implies that the direction of population responses will likely change with contrast, as observed in prior work (9). Extending the model to capture how the magnitudes and the directions of population change during adaptation is the next natural step in this line of research.

## DATA AVAILABILITY

Data including the mean responses of the population for each experiment can be found in the Figshare repository at https://doi.org/10.6084/m9.figshare.25042007.

## SUPPLEMENTAL DATA

10.6084/m9.figshare.25042007 Supplemental Table S1: https://doi.org/10.6084/m9.figshare.25042007.

## GRANTS

This study was supported by NS116471 and EY034488 to D.L.R. and by EY03564 to M.D. and D.L.R. 

## DISCLOSURES

No conflicts of interest, financial or otherwise, are declared by the authors.

## AUTHOR CONTRIBUTIONS

M.D. and D.L.R. conceived and designed research; E.T. and D.L.R. performed experiments; M.D. and D.L.R. analyzed data; M.D. and D.L.R. interpreted results of experiments; D.L.R. prepared figures; D.L.R. drafted manuscript; M.D. and D.L.R. edited and revised manuscript; E.T., M.D., and D.L.R. approved final version of manuscript.
